# Mechanism and strategies of immunotherapy resistance in colorectal cancer

**DOI:** 10.3389/fimmu.2022.1016646

**Published:** 2022-09-27

**Authors:** Jiqi Shan, Dong Han, Chunyi Shen, Qingyang Lei, Yi Zhang

**Affiliations:** ^1^ Biotherapy Center and Cancer Center, The First Affiliated Hospital of Zhengzhou University, Zhengzhou, China; ^2^ State Key Laboratory of Esophageal Cancer Prevention & Treatment, Zhengzhou University, Zhengzhou, China; ^3^ Henan Key Laboratory for Tumor Immunology and Biotherapy, Zhengzhou, China

**Keywords:** colorectal cancer, immunotherapy, immune checkpoint inhibitors, drug resistance, potential therapeutic strategies

## Abstract

Colorectal cancer (CRC) is the third most common cancer in the world. Although there are standard treatment options for CRC, most patients respond poorly to these treatments. Immunotherapies have gradually emerged due to the increasing awareness and understanding of tumor immunity, exhibiting good therapeutic efficacy in various cancers. Immunotherapies include cytokines, immune checkpoint inhibitors (ICIs), and adoptive cell therapies. In particular, ICIs, which are antibodies against cytotoxic T lymphocyte-associated protein 4 (CTLA-4), programmed cell death 1 (PD-1), or its ligand PD-L1, have been successfully applied clinically for solid tumors, relieving the inhibitory effect of the tumor microenvironment on T cells. However, only a minority of patients with cancer achieve a durable clinical response during immunotherapy. Several factors restrict the efficacy of immunotherapy, leading to the development of drug resistance. In this review, we aimed to discuss the current status of immunotherapy for CRC and elaborate on the mechanisms that mediate resistance to immunotherapy and other potential therapeutic strategies.

## Introduction

Colorectal cancer (CRC) has a high morbidity rate and poor prognosis. The five-year survival rate for patients with advanced CRC is around 14%, and metastasis occurs in more than 50% of patients with CRC (1, 2). Immunotherapies comprise a novel and effective therapeutic strategy for patients with various cancers. With recent developments in cancer immunotherapy, both hematological and solid tumors respond to this treatment. In the last decade, immunotherapy has become popular as an alternative to surgery, chemotherapy, and radiotherapy for treating various tumors (3, 4).

Immune checkpoints are a class of molecules expressed on the surface of immune cells that regulate the level of immune activation. They prevent autoimmune abnormalities and launch immune attacks on normal cells. However, in tumors, immune checkpoints, such as programmed cell death 1 (PD-1) and cytotoxic T lymphocyte antigen 4 (CTLA-4), are abnormally activated, resulting in a weakened tumor immune response (5–7). As a result of its ability to interfere with the interaction between immune checkpoints and their receptors, immune checkpoint blockade (ICB) therapy has shown impressive therapeutic effects on a wide variety of tumor types. For CRC, two PD-1-blocking antibodies, pembrolizumab and nivolumab, which are already approved by the Food and Drug Administration (FDA), have shown efficacy in patients with mCRC(metastatic colorectal cancer) that are mismatch-repair-deficient and have high microsatellite instability (dMMR–MSI-H). The ICB drug ipilimumab, a fully humanized monoclonal antibody, blocks the CTLA-4 receptor and has also been approved by the FDA in combination with nivolumab to treat dMMR-MSI-H CRC (8–10). In a study of patients with locally advanced mismatch repair-deficient (dMMR) CRC, 14 patients achieved complete clinical remission after six months of treatment with the anti-PD-1 drug dostarlimab-gxly alone. The exciting result is that dostarlimab has saved all patients in that study from chemotherapy, radiation, or surgery (11). However,immunotherapy is effective in some cases of dMMR-MSI-H CRC but is minimally effective in pMMR-MSI-L CRC. Many colon cancer patients show resistance to immunotherapy. Hence, improving the efficacy of immunotherapy for dMMR-MSI-H and exploring new mechanisms for the treatment of pMMR-MSI-L CRC is key to improving the prognosis of patients with tumors.

## Immunotherapy in CRC

### Immunotherapy in dMMR–MSI-H and pMMR–MSI-L CRC

Colorectal cancer can be classified through a mismatch repair/microsatellite instability system. Microsatellites represent a kind of tandem repeats including 1–6 nucleotides that frequently occur in nuclear genomes (12). Microsatellite instability (MSI) refers to the insertion/deletion of repeated DNA nucleotide units in microsatellites. During DNA replication, the mismatch repair (MMR) system corrects insertions, deletions, or mismatched bases and identifies and repairs DNA damage (13). MMR deficiency results in the failure to detect and correct microsatellite replication errors, resulting in diffuse MSI. High microsatellite instability usually gives rise to the accumulation of somatic mutations. Of these, frameshift mutations are highly positively correlated with the frequency of neoantigens, MSI, and MMR deficiency. They are usually detected in CRC *via* immunohistochemistry as the loss of MMR proteins (MSH2, MSH6, MLH1 and PMS2) or by testing MSI *via* PCR (14, 15). Based on the mutations of MMR proteins and MSI, CRC can be classified into three main types: dMMR-MSI-H tumors with a higher overall mutation burden (>12 mutations per 10^6^ DNA bases), pMMR–MSI-L tumors with a much lower mutation burden (<8.24 mutations per 10^6^ DNA bases), and pMMR-MSS tumors lacking MSI features (13, 16). The MSS/MSI-L subtypes occupy a large proportion (85%) of CRC cases, whereas the dMMR/MSI-H patients accounts for only approximately 15% of all CRC cases and 5% of the mCRCs (17).

**Figure 1:**
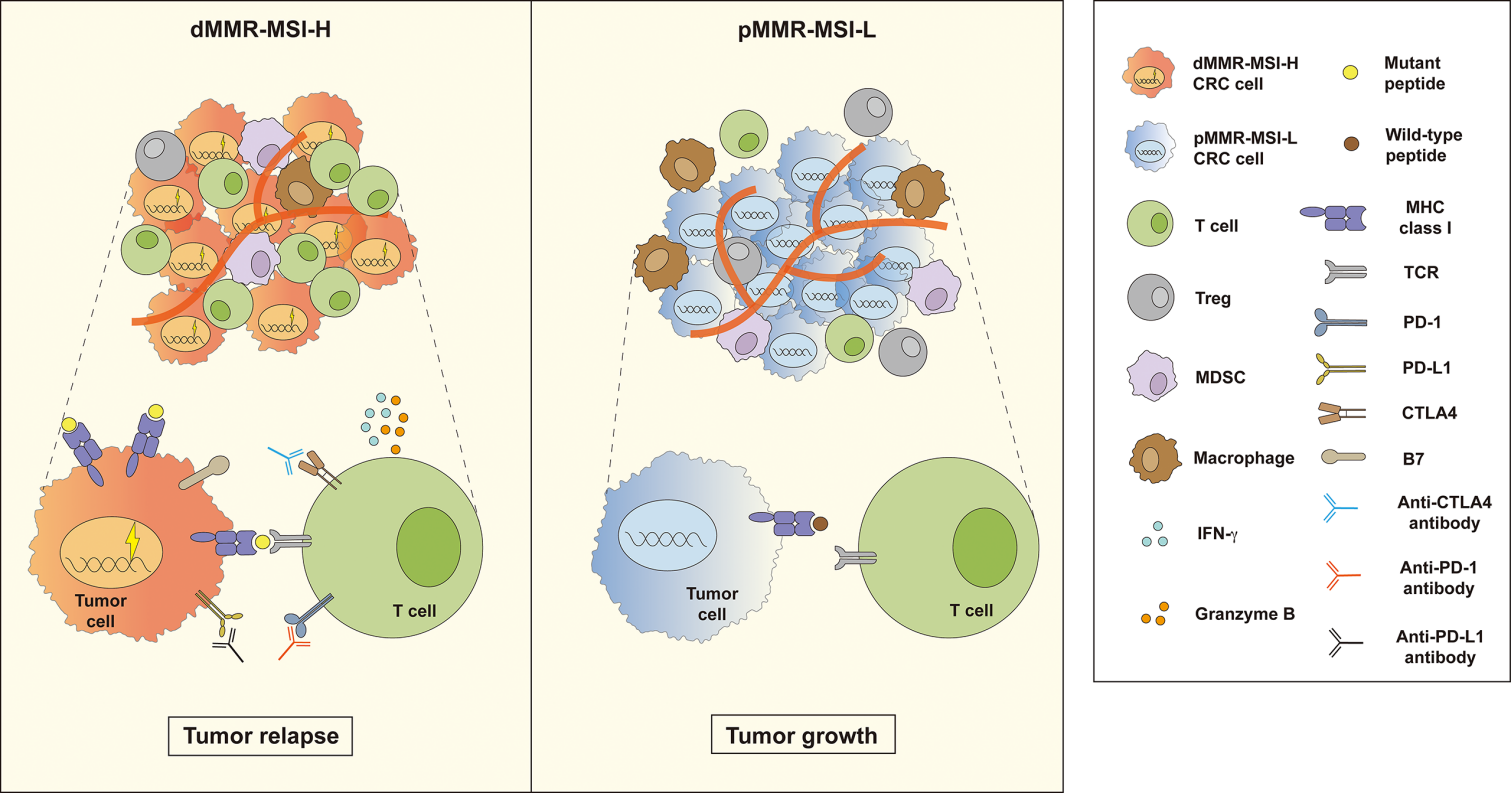
Two outcomes of immunotherapy in dMMR–MSI-H and pMMR–MSI-L CRC. Compared with pMMR–MSI-L, patients with dMMR–MSI-H experience better tumor reduction after treatment with immune checkpoint inhibitors (ICIs). Many functional tumor infiltrating lymphocytes (TILs) release a large number of cytokines such as IFN-γ and granzyme B in the dMMR–MSI-H tumor microenvironment (TME). However, the TME of pMMR–MSI-L CRC contains fewer functional TILs and more immunosuppressive cells, such as Tregs, MDSCs, and TAMs, which inhibit TIL function.

Immune cell function and classification are key factors in immunotherapy, in which tumor-infiltrating lymphocytes (TILs), as the main force of adaptive immune response, play a pivotal role in defense against tumors (18, 19). And powerful immune cytolytic activity (CYT) badgers deeply with many factors including tumor mutation burden and deregulated immune checkpoint (20, 21). Compared to those harboring pMMR-MSI-L/MSS, dMMR-MSI-H patients usually showed satisfactory prognosis and responded better to ICIs, since they owned a high mutational burden whose accumulation might produce more neoantigens for immune recognition. Because of the higher neoantigen load, dMMR–MSI-H tumors are usually heavily infiltrated by functional TILs, which quickly start it activation program and release a large number of cytokines when receiving stimulus from antigen-presenting cells (4, 22). Furthermore, it proves that MSI-CRC has higher expression levels of immune checkpoints, such as PD-L1, CTLA-4 as well as LAG-3, compared with MSS-CRC, which may explain the positive response of dMMR–MSI-H subtype to immunotherapies (18). A phase 2 clinical study (NCT03206073) confirmed that patients with MSI-CRC show much better progression-free survival (PFS) and objective response rate (ORR) than MSS ones when treated with pembrolizumab. Nevertheless, there are few functional TILs and many immunosuppressive cells, consisting of Tregs, MDSCs, and TAMs, infiltrated in the pMMR-MSI-L tumor microenvironment (**Figure 1**).

### Clinical trials on immunotherapies in CRC

Multiple clinical trials have been conducted to estimate the effects of inhibiting PD-1, PD-L1, or CTLA-4 in dMMR–MSI-H and pMMR–MSI-L CRC. NCT02460198 is a phase II study conducted on 124 previously treated patients with locally advanced unresectable or metastatic dMMR/MSI-H CRC (cohort A: standard therapies including fluoropyrimidine, oxaliplatin, and irinotecan; cohort B: fluoropyrimidine + oxaliplatin or fluoropyrimidine + irinotecan, with or without anti-VEGF/EGFR monoclonal antibody) (23). Pembrolizumab (200 mg) was intravenously administrated to these patients every three weeks until approximately 52 cycles. ORR was the primary endpoint, and disease control rate, PFS, overall survival (OS), tolerability, safety and response duration were the secondary endpoints. The results showed that the ORR was 32.8% (95% CI, 21.3% to 46%) and 34.9% (95% CI, 23.3% to 48.0%) in cohorts A and B, respectively. The median PFS was 2.3 months (95% CI, 2.1 to 8.1 months) and the median OS was 31.4 months (95% CI, 21.4 to 58.0 months) for cohort A. In contrast, the median PFS was 4.1 months (95% CI, 2.1 to 18.9 months) and the median OS was 47.0 months (95% CI, 19.2 months to not reached) for cohort B. Although most of the patients suffered different adverse events during the trial, only five patients withdrew from either cohort because of adverse events. Finally, pembrolizumab proved effective and safe in dMMR/MSI-H CRCs. Compared with blocking PD-1, the effectiveness of strategies to block PDL1 or CTLA-4 is subtle and seems to be weaker in clinical trials. Another phase II trial (NCT02870920) utilizes durvalumab combined with tremelimumab for treating patients with advanced CRC receiving the best supportive care, the treatment group showed a longer OS (median OS: 6.6 months vs. 4.1 months; P-value = 0.07) than the control group (24). However, superior PFS in treatment group was not observed, with a 95% CI ranging from 1.8 to 1.9 months. In addition, the ORR was only 0.8% (one patient) in the anti-PD-L1/CTLA-4 therapy group. ICIs have a significant impact on tumor treatment, nevertheless, only partial patients benefit from this regimen. This motivated us to deepen understanding of tumor immunity and explore new solutions for this disease. To date, the FDA approved three immunotherapeutic drugs between 2017 and 2018 (pembrolizumab, nivolumab, and ipilimumab) to treat MSI-H/dMMR CRC (25). Pembrolizumab has become the first-line regimen for treating CRC cases owning an MSI-H/dMMR or unresectable phenotype. Nevertheless, due to the immunosuppressive TME and the significantly low ratio of the MSI-H/dMMR subset in all patients with CRC, patients show resistance to immunotherapy and obtain limited improvement from these immunotherapies. Thus, developing new immunotherapeutic targets and combined therapeutic strategies has become a hot topic (**Table 1** and **Table 2**). And numerous trials have focused on these issues through different mechanisms, such as remolding the antibody structure (Tislelizumab, KN035), combining with other targets (anti-VEGF) or plus with chemoradiotherapy, etc. For example, KN035 is a novel anti-PD-L1 antibody with remolded structure empowering it with superior solubility and stability, which makes KN035 the first checkpoint inhibitor administered subcutaneously. A single-arm, phase II study NCT03667170 found that KN035 therapy showed a pretty good ORR of 43.1% (95% CI, 30.8% to 56.0%) in a cohort of 65 advanced CRC patients during a 28-day treatment cycle (26). Since most of the newly admitted clinical trials are still in recruiting status, the results of these strategies are in urgent to see.

**Table 1:** Table.

Target	Checkpoint inhibitor	Phases	Study treatment groups	Trial identifier	
**Ongoing trials in dMMR/MSI-H CRC**					
PD-1	Pembrolizumab	Phase 2	Pembrolizumab+Olaparib		NCT05201612
PD-1	Pembrolizumab	Phase 2	Pembrolizumab		NCT04895722
PD-1	Pembrolizumab	Phase 2	Pembrolizumab		NCT03638297
			Pembrolizumab+cox inhibitor		
			Atezolizumab+Bevacizumab+Mfolfox6		
PD-1	Nivolumab	Phase 3	Nivolumab+Ipilimumab+Fluorouracil		NCT04008030
PD-1	Nivolumab+Ipilimumab	Phase 2	Nivolumab		NCT04730544
			Nivolumab+Ipilimumab		
			Chemotherapy		
PD-1	Tislelizumab	Phase 2	Tislelizumab		NCT05116085
PD-L1	KN035	Phase 2	KN035		NCT03667170
PD-L1	Atezolizumab	Phase 2	Atezolizumab		NCT05118724
			Atezolizumab+IMM-101		
PD-L1	Atezolizumab	Phase 3	Atezolizumab		NCT02997228
CTLA-4	Ipilimumab	Phase 1	Ipilimumab		NCT04117087
			Nivolumab		
			KRAS peptide vaccine		
**Ongoing trials in pMMR–MSI-L CRC**					
PD-1	Sintilimab	Phase 1|Phase 2	Sintilimab + XELOX + Bevacizumab		NCT04940546
PD-1	Tislelizumab	Phase 2	Tislelizumab		NCT05160727
PD-1	Pembrolizumab	Phase 1|Phase 2	Pembrolizumab+Ataluren		NCT04014530
PD-L1	Durvalumab	Phase 1|Phase 2	Durvalumab+Yttrium-90 RadioEmbolization		NCT04108481
CTLA-4+PD-1	balstilimab	Phase 1|Phase 2	balstilimab+botensilimab		NCT05205330
	botensilimab				
PD-L1+anti-VEGF	Atezolizumab	Phase 2	Atezolizumab+XELOX + bevacizumab		NCT04659382
	Bevacizumab		Bevacizumab+bevacizumab		
			XELOX		
PD-1+anti-VEGF	Pembrolizumab	Phase 2	Pembrolizumab+Bevacizumab+Capecitabine		NCT03396926
	Bevacizumab				

**Table 2:** Trials using combination immunotherapies for CRC.

Target	Drugs	Phase	Treatment group	Trial Identifier
PD-L1 CTLA-4	Durvalumab Tremelimumab	Phase 1	Durvalumab+Tremelimumab	NCT01975831
PD-1 IDO1	Pembrolizumab INCB024360	Phase 1|Phase 2	Pembrolizumab+INCB024360	NCT02178722
EGFR PD-L1 RAF VEGF HER2 HER2 MEK	Cetuximab Atezolizumab Vemurafenib Bevacizumab Trastuzumab Pertuzumab Cobimetinib Chemotherapy	Phase 2	5-FU/LV+Cetuximab+Vemurafenib Fluoropyrimidine+Atezolizumab+Bevacizumab Trastuzumab+Pertuzumab Atezolizumab+Cobimetinib Fluoropyrimidine+Bevacizumab	NCT02291289
PD-1	Pembrolizumab Chemotherapy	Phase 2	Oxaliplatin+Leucovorin+5FU+Pembrolizumab	NCT02375672
PD-1 CSF1R	Pembrolizumab AMG820	Phase 1|Phase 2	AMG820+Pembrolizumab	NCT02713529
PD-1 VEGFR	Pembrolizumab Cetuximab	Phase 1|Phase 2	Cetuximab+ Pembrolizumab	NCT02713373
PD-L1 CTLA-4	Durvalumab Tremelimumab	Phase 2	Best Supportive Care Best Supportive Care+Durvalumab+Tremelimumab	NCT02870920
PD-L1 CTLA-4	Durvalumab Tremelimumab Radiotherapy	Phase 2	Radiotherapy+ Durvalumab+ Tremelimumab	NCT03122509
PD-1 CTLA-4 MEK	Nivolumab Ipilimumab Binimetinib	Phase 2	Nivolumab+Binimetinib+ Ipilimumab Nivolumab+Binimetinib	NCT03271047
PD-1 BTK	Pembrolizumab Ibrutinib	Phase 1|Phase 2	Pembrolizumab+Ibrutinib	NCT03332498
PD-1	Pembrolizumab Oncolytic virus	Phase 1	Pembrolizumab+Talimogene Laherparepvec	NCT03256344
PD-1 CCR5	Pembrolizumab Maraviroc	Phase 1	Pembrolizumab+ Maraviroc	NCT03274804
PD-1 CXCR1/2	Pembrolizumab Navarixin	Phase 2	Pembrolizumab+Navarixin	NCT03473925
PD-1 GpA33	MGA012 MGD007	Phase 1|Phase 2	MGD007+MGA012	NCT03531632
PD-1 CCR5	Pembrolizumab Vicrivir	Phase 2	Pembrolizumab+ Vicriviroc	NCT03631407

## Mechanisms of immunotherapy resistance

**Figure 2:**
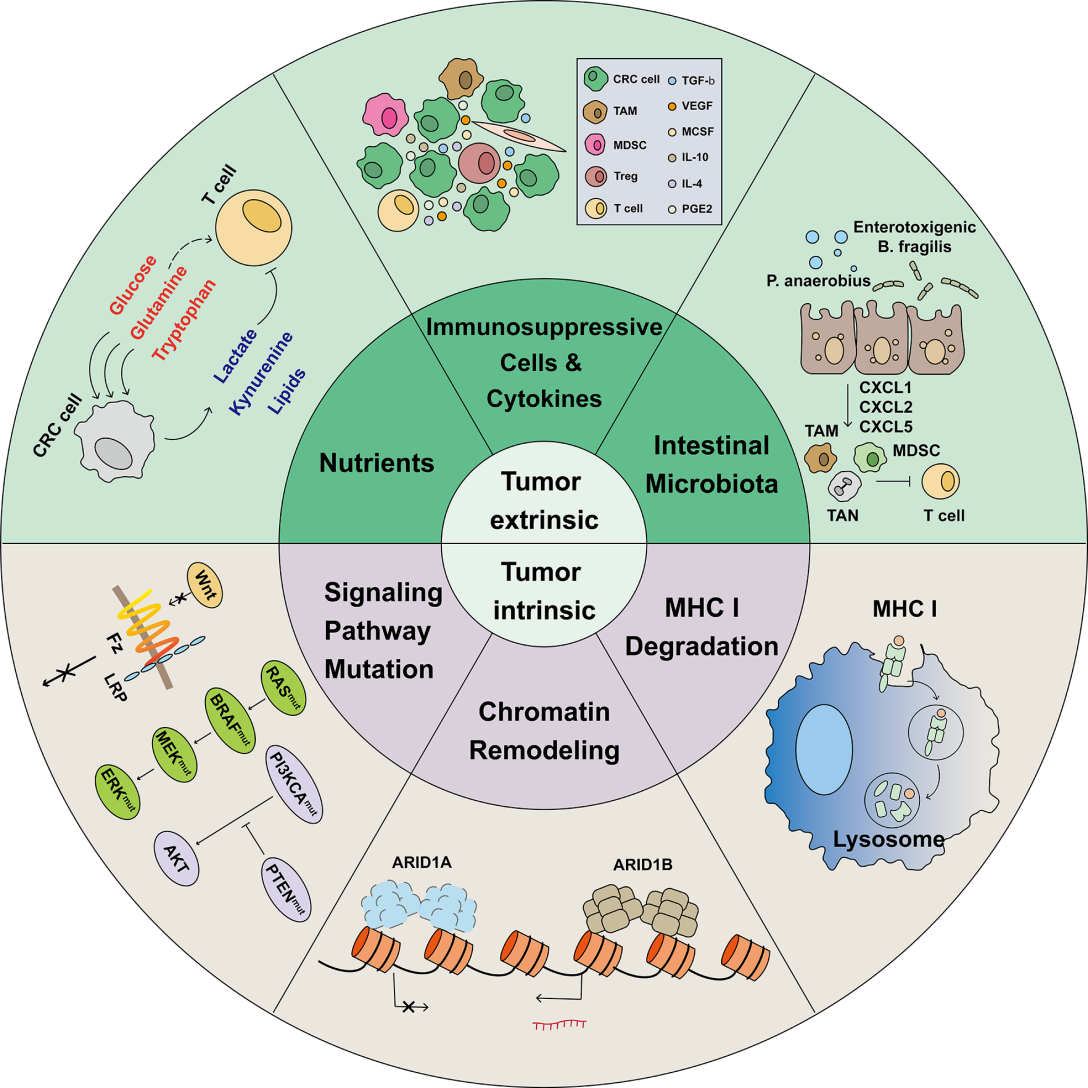
Mechanisms associated with immunotherapy resistance. The limited effectiveness of immunotherapy is primarily due to various mechanisms of immunotherapy resistance. In the heterogeneous tumor microenvironment, cells such as Tregs, MDSCs, and TAMs, combined with tumor-released immunosuppressive cytokines, induce tumor infiltrating lymphocytes (TILs) exhaustion. In the TME, tumor cells have a greater ability to compete for nutrients, such as glucose, glutamine, and tryptophan, which are necessary for proper cellular function. Meanwhile, tumor cells release lactate, kynurenine, and Oxidized low density lipoprotein (oxLDLs), which are harmful to TILs. In CRC, intestinal microbiota such as *P. anaerobius and enterotoxigenic B ragilis* induce tumor cells to release CXCL1, CXCL2, and CXCL5 and recruit immunosuppressive cells. Before identifying and killing TILs, tumor cells activate the lysosomal degradation pathway of MHC class 1 to escape T cell killing. The switching/sucrose non-fermentable (SWI/SNF) complex has been identified as a tumor suppressor gene in CRC; AT-Rich Interactive Domain-containing protein 1A (ARID1A) is the most frequent target of SWI/SNF mutations. However, ARID1A mutations was found correlated with markedly higher level of immune infiltrates in colon cancer. Gene and signal pathway mutations, such as those in WNT, RAS, BRAF, MEK, ERK, PI3KCA, and PTEN, were reported to be associated with immunotherapy resistance.

Multiple mechanisms are involved in the process of immunotherapy resistance. Immunotherapy resistance can divided into Tumor-intrinsic and -extrinsic (**Figure 2**).

### Tumor-extrinsic resistance to immunotherapy in CRC

#### Immunosuppressive cells in the heterogeneous tumor microenvironment

Intercellular interaction is a well-known immunosuppressive mechanism in the TME, with the classic PD-L1/PD-1 signaling between tumor cells and CD8+ T cells being proven in different cancers. Nevertheless, many suppressive cells other than tumor cells also profoundly inhibit T-cell cytotoxicity, including Tregs, MDSCs, and TAMs. These cell subsets differentiate improperly and upregulate immunosuppressant-associated proteins, which usually form an anti-inflammatory environment and result in CD8+ T cell dysfunction and immune resistance.

Regulatory T cells (Tregs) are CD25+ FOXP3+ CD4 T cells originating from the thymus or peripheral blood (27). Tregs function as a tumor-promoting component in the TME because they inhibit the immune response against CRC through cell-to-cell contact or the secretion of cytokines and metabolites. Studies report that Tregs regulate the immune response mainly through several mechanisms (27–29). First, they can secrete cytokines such as IL-10, TGF-β, and IL-35, which facilitate Treg proliferation and suppress effector T cell activity (29–31). In addition, the surface marker CD25, which is the α-chain of the IL-2 receptor, can deprive IL-2 because of its high affinity (32, 33). Second, Tregs can exert anti-inflammatory effects by catalyzing the conversion of pro-inflammatory adenosine triphosphate to anti-inflammatory adenosine through the CD39-CD73 axis (34). Third, Tregs constitutively express immune checkpoints, such as CTLA-4, which impair APC maturation and cause T cell exhaustion (35). Finally, it has been reported that Tregs utilize granzyme and perforin to lyse DCs or T cells (36, 37). Tregs also inhibit T cells from exerting anti-tumor functions in various ways. Hence, thinking about how to reduce Treg populations or inhibit their function is one of the key methods for improving the effect of immunotherapy.

Myeloid-derived suppressor cells (MDSCs) are a heterogeneous population of immature myeloid cells that originate from the bone marrow. The immunosuppressive effects of MDSCs mainly depend on two enzymes: inducible Nitric Oxide Synthase (iNOS) and Arginase 1 (ARG1). High ARG1 catalyzes L-arginine to urea and ornithine,causes a depletion of l-arginine in the TME. iNOS uses l-arginine to produce NO. The depletion of l-arginine impairs T cell proliferation and activity by decreasing CD3ζ expression (38). In addition, NO, in cooperation with O^2-^, can result in T cell apoptosis through the tyrosine phosphorylation of the TCR/CD3 complex (39, 40).

Tumor-associated macrophages (TAMs) are key immune components of the TME. Based on their polarization fate, they can be divided into the M1 (pro-inflammatory) and M2 (anti-inflammatory) phenotypes. Interestingly, M1 and M2 cells can repolarize to each other depending on the interventions administered (for example, by targeting mitochondrial metabolic pathways) (41–43). Numerous studies have reported the supportive effects of TAMs on malignant cells in the CRC TME. TAMs can induce tumor angiogenesis by secreting VEGF and MCP-1, MIP-1α, and MIP-2α to promote tumor growth and invasion (44, 45). TAMs impair T cell function in multiple ways, including restricting infiltration, blocking proliferation and activation, and suppressing cytotoxicity. For instance, IL-10 produced by TAMs can decrease CD8 protein levels and impair TCR signaling (46). Other factors such as iNOS, ARG1, and PD-L1 expression also cause T cell dysfunction. Therefore, transforming TAMs from the M2 phenotype to the M1 phenotype is key to restoring T cell function.

#### Immunosuppressive cytokines in the heterogeneous tumor microenvironment

Cytokines enriched in the TME are another important pathway that interferes with the efficiency of immunotherapy against tumors. Various studies have concluded the vital role of immunosuppressive cytokines (such as TGF-β, VEGF, IL-4, and IL-10) in the disruption of CD8+ T cell function. In brief, immunosuppressive cytokines are well defined to recruit, polarize, or activate immunosuppressive cells and indirectly interfere with T cell function, although some directly inhibit T cell cytotoxicity. These effects restrict the efficiency of immunotherapy.

TGF-β is a multipotent cytokine that inhibits tumor formation at early stages but promotes tumor progression at advanced stages. Most cells in the TME can produce TGF-β, including cancer cells, fibroblasts, TAMs, Tregs, and even platelets (47, 48). Bardeesy et al. reported that TGF-β/SMAD4 signaling results in premalignant pancreatic cell apoptosis harboring KRAS mutations (49). In addition, primary mesenchymal stromal cells in the TME can induce CXCL5 overexpression in CRC, promoting tumor metastasis and angiogenesis *via* the CCL7/CCR1/KLF5 pathway; TGF-β/SMAD4 signaling can reverse this effect (50). However, the pro-tumor and immunosuppressive effects of TGF-β are more important in tumor development and are thought to be potential therapeutic targets. For example, TGF-β enhances EMT in CRC through the USF2/S100A8 axis (51). The TGF-β/SMAD pathway decreases CTL cytotoxicity by decreasing perforin, granzyme A, granzyme B and IFN-γ expression (52). TGF-β is also vital for recruiting suppressive immune cells such as Tregs and TAMs.

Vascular endothelial growth factor (VEGF) is a family of cytokines consisting of five members: VEGF-A, VEGF-B, VEGF-C, VEGF-D, and PlGF. This family of proteins is mainly expressed in CRC tissues and the hypoxic TME and is highly involved in tumor progression and metastasis. The classical function of VEGF is to promote tumor angiogenesis *via* commonly known receptors (VEGFR-1, VEGFR-2, and VEGFR-3) expressed on adjacent vascular endothelia (53, 54). Despite their angiogenic effect, they also strongly influence inflammatory cell infiltration and function. A recent study showed that VEGF-A directly influences T cell exhaustion by inducing the expression of the transcription factor TOX in T cells in MSS CRC (55). Anti-VEGF therapy can also indirectly augment the ability of CD8+ T cells to produce IFN-γ and TNF-α by modulating the hypoxic TME (56).

IL-4 and IL-10 are cytokines classically expressed by Th2 T lymphocytes and are involved in infection and autoimmune diseases. However, they are also highly enriched in multiple cell types in the CRC TME (42, 57–60). Both are important pro-tumor and immune regulatory factors that target IL4R and IL10R receptors, respectively. Koller, F. L. et al. and Liu, H. et al. reported that IL4 could promote HT-29 cell proliferation (61, 62), while Mantilla-Rojas, C. et al. reported that IL-10/SRC contributes to CRC progression (63). IL-4 and IL-10 can suppress CD8+ tumor-infiltrating T cell function by recruiting immunosuppressive components, such as Tregs, M2 TAMs, and MDSCs. However, IL-10 has recently been reported to enhance the anti-tumor effects of CD8+ T cells directly and indirectly (64, 65).

#### Intestinal microbiota

As luminal tract organs communicate with the outside environment, the colon and rectum are exposed to more than 100 trillion microorganisms, including bacteria, fungi, protozoa, and viruses, from their proximal to distal ends. Physically, the gut microbiota is vital for host homeostasis by providing important nutrients such as vitamins and essential amino acids. However, dysbiosis facilitates many pathological processes such as inflammation, dysplasia, and cancer (66–68). Patients with CRC showed significantly different abundances and reduced diversity of the gut microbiota. For example, *Fusobacterium nucleatum* and *Peptostreptococcus anaerobius* are highly enriched in CRC tissues; however, *Clostridium butyricum* and *Bifidobacterium animalis* are depleted (66, 69). The gut microbiota and its metabolites are widely known to participate in tumor growth and immune responses. *F. nucleatum* promotes HCT116 and LoVo cell proliferation by activating TLR4 signaling (70). It polarizes TAMs to the M2 phenotype through diverse pathways contributing to CRC progression and metastasis (71, 72). The Fap2 protein produced by *F. nucleatum* hinders T cell activation by interacting with TIGIT (73). On the contrary, probiotics, a huge commensal bacterial family in the intestine, are of great importance for tumor regression and improving the effects of immunotherapies. *Clostridium butyricum* releases butyrate into the TME and hinders CRC proliferation by inhibiting HDAC activity (74, 75). Recent studies have shown that the administration of *Lactobacillus rhamnosus GG* and *Lactobacillus casei* can facilitate CD8+ TIL infiltration and cytotoxic cytokine secretion, improving the anti-tumor effect of anti-PD-1 agents (76, 77). As a newly identified TME factor in CRC, the influence of the microbiota on tumor cells and CD8+ T cells is complex and requires further research. Patients with CRC generally show an unbalanced bias towards pro-tumor microbiota enrichment, which indicates that recovering intestinal microbiota homeostasis or the exogenous administration of anti-tumor microbiota and its metabolites is closely related to the prognosis associated with CRC immunotherapy.

#### Nutrients in the heterogeneous tumor microenvironment

Altered nutrient metabolism is a hallmark of many tumors. As members of the hypoxic and resource-limited TME, tumor cells and CD8+ T cells urgently require large amounts of nutrients to maintain their survival and biological activities. Tumor cells usually exhibit higher glucose consumption and stronger competitive uptake of essential amino acids, exacerbating the shortage of important nutrients and hindering T cell survival and cytotoxicity. In addition, increased lipid production and the generation of transformation products from glucose and some amino acids are also critical immunosuppressive factors that impair T cell immune responses.

Distinct from the normal colorectal epithelium, CRC cells consume large amounts of glucose to maintain rapid expansion. However, they are prone to glycolysis rather than OXPHOS even in ample oxygen conditions, classically called the Warburg effect. In this process, glucose is converted into pyruvate and ATP together with an important pro-tumor byproduct, lactate, which further contributes to acidosis in the CRC TME (78, 79). Different mechanisms have been reported to regulate glucose uptake by CRC cells. For example, Tang et al. and Wang et al. concluded that the lncRNAs GLCC1 and LINRIS promote tumor glycolysis and facilitate CRC progression by stabilizing c-MYC transcription in mouse or PDX models, correlating with poor prognosis in patients with CRC (80, 81). Moreover, enhanced glycolysis also induced the resistance of CRC cells to chemotherapies such as 5-FU (82). In addition to competitive uptake, malignant cells can directly impair glucose metabolism in T cells by expressing CD155 and combining with TIGIT on the T cell surface, resulting in dysfunction in T cell energy utilization (83–85). The byproduct lactate is also a versatile factor that promotes CRC progression and metastasis through various mechanisms. Deng et al. recently reported that lactate stimulates the tube formation of endothelial cells and eventually angiogenesis in the CRC TME (86). Moreover, several articles have reported the effect of lactate on M2 phenotype polarization in TAMs (87–89). Therefore, eliminating lactate and improving the acidic TME restore CD8+ T cell anti-tumor immunity in many cancers (90–92).

Tryptophan is an essential amino acid that participates extensively in tumor immunity in different cancer types. Three enzymes, IDO1, IDO2, and TDO2, are responsible for tryptophan catabolism, transforming more than 95% of tryptophan to kynurenine, leading to the deficiency of tryptophan in the TME (93, 94). The transformed product, kynurenine, is a profoundly immunosuppressive metabolite. Tumor-derived kynurenine induces PD-1 expression in CD8+ T cells by activating the transcription factor AHR both in a mouse model and in patient samples (95). In addition, kynurenine is negatively correlated with CD8+ T cell infiltration in the CRC TME. The induction of FOXP3 and Treg polarization by kynurenine may contribute to this process (96, 97).

Unlike tryptophan, glutamine is a nonessential amino acid that is highly consumed in many malignancies. It renders many vital biological processes, including nutrient exchange and the biosynthesis of other amino acids, lipids, and nucleotides, by providing nitrogen and carbon (98, 99). As the second most abundant nutrient after glucose in the TME, glutamine catabolism is an indispensable method of energy generation *via* the TCA cycle (99, 100). Because of its role as a substrate for many bioactive elements and ATP production, glutamine is required in malignant cells and other immune components. Turowski et al. first confirmed the ability of glutamine to stimulate the proliferation of human colon cancer cell lines Caco-2 and SW620 (101). Further studies have shown that KRAS mutations heighten glutamine uptake in CRC cells by upregulating SLC1A5 expression. Moreover, it was found that glutaminolysis-derived succinate promotes CRC cell proliferation and stemness by upregulating LRG5 expression and enhancing Wnt/β-catenin signaling (102, 103). Lack of glutamine is seriously affect T cell proliferation after activation (104).

Lipids are a family of hydrophobic or amphipathic molecules that consist of fatty acids, glycerophospholipids, sphingolipids, and sterols. Although high lipid accumulation in the CRC TME has been recognized, their profound impact on tumor-infiltrated CD8+ T cells has only been noticed in recent years. CRC cells can produce various lipids, such as fatty acids, obliging T cells to uptake these lipids, resulting in T cell dysfunction (105–107). Mechanistically, CD8+ T cells in the TME showed the increased expression of CD36, a scavenger receptor for fatty acid uptake. The abnormal accumulation of fatty acids inside T cells further initiates lipid peroxidation, induces T cell ferroptosis, and reduces the production of cytokines, such as IFN-γ and TNF-α (107).

### Tumor-intrinsic resistance to immunotherapy in CRC

#### MHC degradation

The T cell antigen receptor (TCR) is a multimolecular structure expressed on the T cell membrane that recognizes peptide/MHC complexes on the tumor cell surface (108). The expression of MHC class I molecules is crucial for an effective adaptive immune response. A low expression or deletion of MHC class I molecules frequently occurs in colon tumors resistant to immunotherapy (109–111). The oncoprotein SND1 promotes the ER-associated degradation of MHC class I, resulting in disordered CD8+ T cell function and decreased anti-tumor ability (112). A rational combination of systemic chemotherapy and DC i.t. injection has been shown to induce a complete anti-tumor response in MC38 murine adenocarcinoma cells (113). Moreover, increased mitophagy in intestinal epithelial cells can trigger lysosomal membrane permeability, and the subsequent release of proteases into the cytoplasm increases MHC class I presentation and activation of CD8+ T cells *via* cross-modification of dendritic cells (114).

#### Gene mutations

The occurrence and development of CRC result from multiple gene interactions and the involvement of various signaling pathways. However, gene mutations and defects in signaling pathways also affect the immunotherapy sensitivity and prognosis of patients with colon cancer (115). As a key cascade that regulates cell development and stemness, Wnt/β-catenin pathway plays a prominent role in the occurrence of colon cancer (116). The aberrant activation of the Wnt/β-catenin pathway is a key driver in the maintenance and proliferation of gastrointestinal stem cells (117). The Wnt/β-catenin signaling pathway is also considered an important carcinogenic signaling pathway related to immune evasion. Activation of Wnt/β-catenin pathway in tumor leads to the decreasing production of CCL4 released by CD103+ dendritic cells. This inhibit CD8+ T cell activation and infiltration (118, 119). The Cancer Genome Atlas Network found that 55% of non-hypermutated tumors had alterations in KRAS, NRAS, or BRAF, which have significant mutually exclusive mutation patterns (12). PIK3CA mutations are some of the most common genetic alterations in solid tumors, inducing defects in the phosphatidylinositol 3-kinase (PI3K)/mammalian target of rapamycin (mTOR) pathway, which is frequently dysregulated. PIK3CA mutations lead to the attenuation of tumor apoptosis and improvement of tumor invasion. Treatment with the PI3K inhibitor LY294002 has been shown to downregulate PIK3CA signaling and inhibit the progression of PIK3CA-mutant colon cancer (120). Meanwhile, mutations of PIK3CA could increase total mutation burden (TMB) which may improve immunotherapy sensitivity (121).

#### Chromatin remodeling (SWI/SNF complex)

The switching/sucrose non-fermentable (SWI/SNF) complex regulates transcription *via* nucleosome topology modulation and has been identified as a cancer suppressor gene in human malignancies (122, 123). SWI/SNF exhibits a broad mutation pattern associated with the progression and invasion of tumor cells. AT-Rich Interactive Domain-containing protein 1A (ARID1A), as one of the components making up the largest SWI/SNF subunit together with AT-Rich Interactive Domain-containing protein 1B (ARID1B), is the most frequent target of SWI/SNF mutations. mutations in ARID1A impair enhancer-mediated gene regulation and prognosis in patients with colon cancer (124, 125). Interestingly, SWI/SNF complex stability is also vital for tumor cell viability, which might be impaired by ARID1A mutation. In that situation, ARID1B is usually functionally normal and enable to partially compensate for ARID1A function as its homolog. But simultaneous mutation of ARID1A and ARID1B is synthetically lethal for colon cancer. ARID1A mutations was found correlated with markedly higher level of immune infiltrates in colon cancer. MSH2, a Mismatch repair protein, was found to interact with ARID1A. Low expression of ARID1A impair DNA mismatch repair, resulting in increases in TMB and cytotoxic T cell infiltration (126).

## Novel strategies to overcome immunotherapy resistance

**Figure 3:**
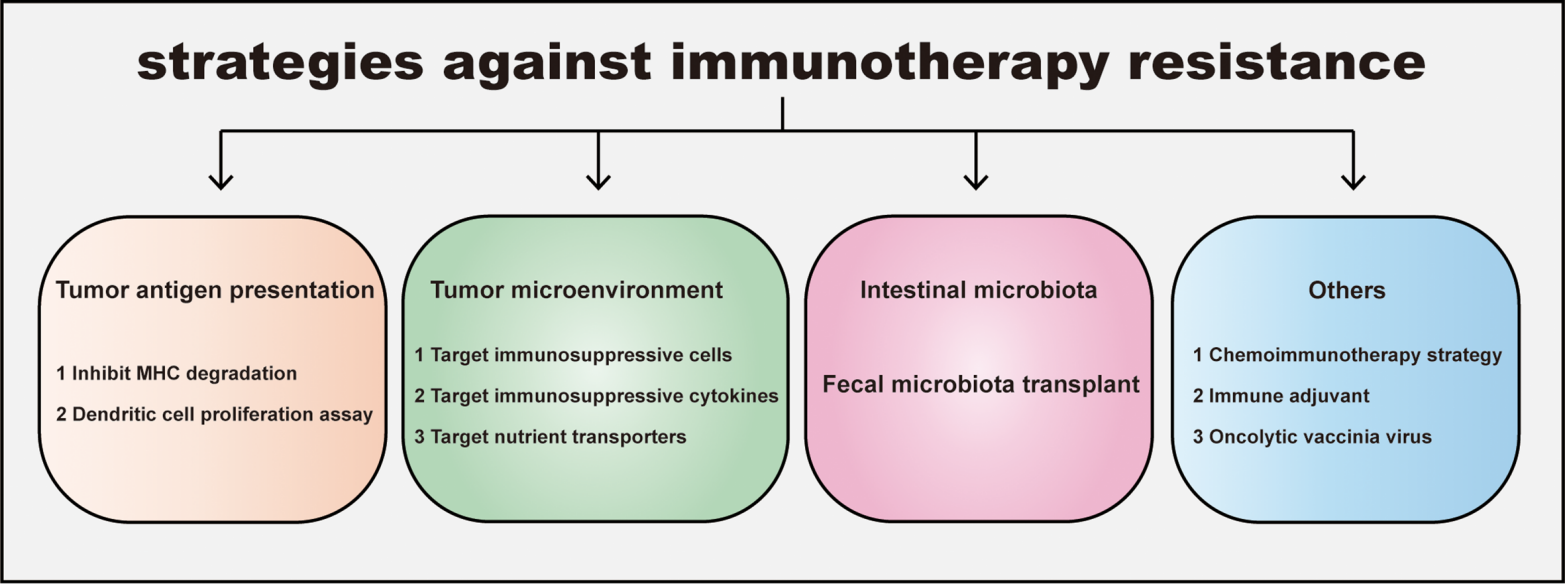
Strategies for overcoming immunotherapy resistance in CRC. The resistance leads to an ineffective immunotherapy and tumor progression. Four distinct strategies against immunotherapy resistance are listed: promoting tumor antigen presentation, regulating tumor immunosupprssive microenvironment, manipulating intestinal microbiota and others including combination therapy etc. Inhibiting MHC degradation and boosting dendritic cell proliferation could elevate tumor antigen presentation; Targeting immunosuppressive cells, cytokines in Tumor microenvironment (TME) and nutrient transporters help CD8+T escape the inhibition of TME. In addition, gut microbiota and its metabolite are widely proven to participate in tumor growth and immune response. Consistantly, fecal microbiota transplantation makes patients sensitive to immunotherapy. Other strategies for overcoming immunotherapy resistance, such as the administration of chemoimmunotherapy, immune adjuvant, oncolytic vaccinia virus can also improve the sensitivity of immunotherapy in CRC.

Immunotherapy resistance of decrease immunotherapy sensitivity in CRC. Targeting these tumor intrinsic and extrinsic resistance mechanisms is the key to improving the effectiveness of immunotherapy (**Figure 3**).

### Chemoimmunotherapy strategy

Chemotherapy was previously thought to be immunosuppressive due to its concomitant severe myelosuppression and leukopenia (127). However, numerous studies have reported its synergistic effects to immunotherapy, which makes the chemoimmunotherapy come back to clinical practice and become standard care for selected patients (128–130). Indeed, chemotherapy is found to induce tumor immunogenic cell death (ICD), which elicits extensive innate and adaptive immune response. For example, the classical chemotherapy regimen FOLFOX or FOLFIRI is reported to induce DAMPs (damage‐associated molecular patterns) in both mouse and human tumor cell lines. And DAMPs is a strong signaling to promote DC maturation and tumor antigen presentation, thus leading to tumor ICD and subsequent enhanced T cell anti-tumor response (131). According to the current conclusions, seeking the dose balance of chemotherapy and immunotherapy may be an attractive strategy to avoid the side effects and boost the therapeutic effects. To illustrate the combined efficacy of immunotherapy and chemotherapy in colorectal cancer, NCT02375672 administrated Pembrolizumab (MK-3475) together with standard-dose mFOLFOX6 regimen to treat 30 advanced CRC patients. And the median PFS is 8.8 months (95% CI, 7.7 to 11.3 months) with ORR being 56.7%, which showed exciting potential of combined medical strategies (132).

### Dendritic cell proliferation assay

Before enhancing T cell function with various exogenous elements, the first step for T cells in killing tumor cells is to recognize tumor antigen epitopes, in which endogenous DCs play a key role. However, due to insufficient DC infiltration and abnormal maturation affected by the TME, tumor antigens are usually not well-presented. Thus T cells cannot differentiate and be activated normally. Thus, promoting DC expansion and maturation is a promising method for boosting T cell-based therapy. Fms-like tyrosine kinase 3 ligand (Flt3L) is a growth factor that expands dendritic cells, increasing their number (133). A recent study developed a strategy called an *in situ* vaccine, which combined Flt3L, radiotherapy, and a TLR3 agonist (Flt3L for DC expansion, radiotherapy for loading DCs with the antigen, and TLR3 agonist for DC activation) to test its efficacy in a murine lymphoma model (134). Mechanistically, this strategy expanded and activated intra-tumoral DCs, which further promoted TAA presentation and anti-tumor T cell activation. With an exciting long-term tumor regression of at least 3 months, researchers conducted a clinical trial on patients with low-grade B-cell lymphoma using rhuFlt3L and poly-ICLC combined with low-dose radiotherapy. Preliminary results showed good tolerance and an ORR of 72.7%. This has aroused our interest in the relevance of dendritic cells to ICB therapy and revealed the possibility of developing novel therapies that effectively control drug-resistant CRC, such as the evaluation of combinations of Flt3L and ICIs in clinical trials.

### Tumor microenvironment

Targeting the TME is another potential way to boost the effect of immunotherapy. For example, a clinical trial (NCT04126733) that began in 2019 focused on the efficacy of regorafenib, a VEGFR inhibitor targeting TME angiogenesis, in combination with nivolumab in patients with pMMR-MSS CRC. The primary endpoint ORR was only 7.1% (95% CI, 2.4% to 15.9%), and the median PFS was 8.00 weeks (95%CI, 7.86 to 10.57 weeks). In another trial (NCT02713529) that used pembrolizumab combination with AMG820, a CSF1R inhibitor repolarizing TMAs from M2 to M1 type (135), the ORR for patients with pMMR CRC was quite low (4.9%; 95% CI, 0.60% to 16.53%).

### Fecal microbiota transplants

Dysbiosis in CRC has multiple influences on the TME, which establishes an environment that favors tumor immune escape. Since patients with tumors always harbor altered microbiota characteristics, rebuilding intestinal microbiota homeostasis may be an attractive way to overcome treatment resistance in these patients. One plausible method is to transplant the microbiota of immunotherapy responders to non-responders. In the clinical trial NCT03341143 that tested this hypothesis, researchers carefully screened two patients with metastatic melanoma whose tumors had completely disappeared after prior PD-1 therapy (136). Then transplant the microbiota of screened two patients to non-responders.Miraculously, the recipients showed tumor reduction or stable disease for more than a year, with an ORR of 20% and a median PFS of 3 months (median follow-up of 7 months). Microbiota transplantation reversed the response to PD-1 drugs in patients with anti-PD-1-refractory melanoma (137, 138). This reveals the potential value and significance of gut microbiota in immunotherapy. In addition, through the adjusted daily diet and the intake of prebiotics, the gut microbiota can influence the pre-existing commensal microbes in the gut. In the future, improving the gut microbiota *via* gut microbiota transplantation or other methods is likely to improve the effectiveness of immunotherapy in patients with CRC.

### Oncolytic viruses and engineered bacteria

Due to the low immunogenicity of various tumor cells and the immunosuppressive TME, natural T cells usually have a limited anti-tumor response even in the presence of ICIs. Engineered chimeric antigen receptor T (CAR-T) cells armored with various molecules have recently been developed to resolve this issue (134). Nevertheless, the medical efficacy of CAR-T therapy is still limited because of the low recruitment ratio and the presence of only a few desirable epitopes (139, 140). The use of oncolytic viruses (OVs) and engineered bacteria are promising methods to overcome the deficiencies of CAR-T cells because not only can they get into tumor tissues unimpeded and directly kill tumor cells, but they can also release or amplify tumor immunogenicity to stimulate immune cell responses, enhance immune cell infiltration, and result in effective anti-tumor immunity (141). A recent study reported that an oncolytic virus called CD19t could induce tumor cells to express CD19 on their surface before killing them, subsequently redirecting CD19-CAR-T cytotoxicity and enhancing the anti-tumor response in mouse models (132). In another research of murine CRC model, a kind of engineered Brucella melitensis (BmΔvjbR) in short of virulence was developed to assist CAR-T efficacy. The team found that intravenous administration of BmΔvjbR could significantly promote intratumoral M1-macrophage polarization and expansion, which further enhanced CAR-T infiltration and activity (142). However, no studies have focused on combining OVs/engineered bacteria and CAR-T cells in clinical patients, which is an urgent need.

### Immunoadjuvants

Immunoadjuvants are non-specific non-immunogenic immunostimulatory molecules that can elicit rapid and strong immune responses when administered before or simultaneously with pathogen antigens. However, currently proven clinical immunoadjuvants are usually highly involved in activating humoral immunity instead of cellular immunity, limiting their application mostly in bacterial and some viral infections, like in various vaccines (140). This provoked our interest in seeking adjuvants that prefer the activation of cellular immunity to treat tumors since T cells play a key role in the anti-tumor response. Fortunately, recent studies have shown that manganese salt is an excellent adjuvant in eliciting both NK and T cell anti-tumor responses that mainly promoted DC maturation and antigen cross-presentation in preclinical models, a major breakthrough in the field. In particular, combination therapies comprising anti-PD-1 agents and manganese chloride have shown great preliminary clinical efficacy, with a median ORR of 45.5% in a phase I clinical study, NCT03991559 (143). Thus, manganese salt administration is anticipated for the future development of immunotherapies and tumor vaccines.

## Conclusions

The number and function of TILs determine tumor prognosis in tumor immunotherapy. Hence, improving the number and function of TILs is the key to addressing immunotherapy resistance. By further understanding the heterogeneity of the tumor microenvironment and the defense and escape mechanisms of the gut microbiota and tumor cells against the attack of the immune system, we can target these drug resistance mechanisms to reduce immunotherapy resistance and improve the prognosis of patients with cancer. It is believed that with ICIs as the primary therapeutic backbone, targeting the tumor microenvironment and gut microbiota using combination treatments comprising ICIs, radiotherapy, chemotherapy, and various new therapeutic modalities will be continuously carried out, resulting in a higher percentage of patients that would benefit from immunotherapy.

## Author contributions

JS and DH contributed to conception and design of the study. JS organized the database. JS, DH and CS wrote the manuscript and created the figures and tables. JS, DH and QL wrote sections of the manuscript. All authors contributed to the article and approved the submitted version.

## Funding

This work was funded by National Natural Science Foundation of China (grant nos. 81872333, U1804281, 91942314), Henan Provincial Medical Science and Technology Research Plan Provincial and Ministerial Joint Construction Project (grant no. SBGJ202101010).

## Conflict of interest

The authors declare that the research was conducted in the absence of any commercial or financial relationships that could be construed as a potential conflict of interest.

## Publisher’s note

All claims expressed in this article are solely those of the authors and do not necessarily represent those of their affiliated organizations, or those of the publisher, the editors and the reviewers. Any product that may be evaluated in this article, or claim that may be made by its manufacturer, is not guaranteed or endorsed by the publisher.
